# DEHP Decreases Steroidogenesis through the cAMP and ERK1/2 Signaling Pathways in FSH-Stimulated Human Granulosa Cells

**DOI:** 10.3390/cells12030398

**Published:** 2023-01-22

**Authors:** Biljana Tesic, Dragana Samardzija Nenadov, Tamara Tomanic, Svetlana Fa Nedeljkovic, Stevan Milatovic, Bojana Stanic, Kristina Pogrmic-Majkic, Nebojsa Andric

**Affiliations:** 1 Department of Biology and Ecology, Faculty of Sciences, University of Novi Sad, 21000 Novi Sad, Serbia; 2 Clinical Center of Vojvodina, Clinic for Gynecology and Obstetrics, Faculty of Medicine, University of Novi Sad, 21000 Novi Sad, Serbia

**Keywords:** DEHP, human granulosa cells, estradiol, progesterone, cAMP, ERK1/2

## Abstract

DEHP is an endocrine disruptor that interferes with the function of the female reproductive system. Several studies suggested that DEHP affects steroidogenesis in human and rodent granulosa cells (GC). Some studies have shown that DEHP can also affect the FSH-stimulated steroidogenesis in GC; however, the mechanism by which DEHP affects hormone-challenged steroidogenesis in human GC is not understood. Here, we analyzed the mechanism by which DEHP affects steroidogenesis in the primary culture of human cumulus granulosa cells (hCGC) stimulated with FSH. Cells were exposed to DEHP and FSH for 48 h, and steroidogenesis and the activation of cAMP and ERK1/2 were analyzed. The results show that DEHP decreases FSH-stimulated STAR and CYP19A1 expression, which is accompanied by a decrease in progesterone and estradiol production. DEHP lowers cAMP production and CREB phosphorylation in FSH but not cholera toxin- and forskolin-challenged hCGC. DEHP was not able to decrease steroidogenesis in cholera toxin- and forskolin-stimulated hCGC. Furthermore, DEHP decreases FSH-induced ERK1/2 phosphorylation. The addition of EGF rescued ERK1/2 phosphorylation in FSH- and DEHP-treated hCGC and prevented a decrease in steroidogenesis in the FSH- and DEHP-treated hCGC. These results suggest that DEHP inhibits the cAMP and ERK1/2 signaling pathways, leading to the inhibition of steroidogenesis in the FSH-stimulated hCGC.

## 1. Introduction

Di-2-ethylhexyl phthalate (DEHP) is the most common member of the phthalates, a group of chemicals widely used as plasticizers in polymer products to make plastic flexible [1]. DEHP is present in many medical devices, such as intravenous bags and tubing, umbilical artery catheters, and nasogastric tubes, and it is also utilized in the manufacturing of a wide variety of consumer products, such as packed food and beverages [2], toys [3], building and furniture materials [4], and cosmetics [5]. Due to its extensive use and leaching out of plastic products, DEHP readily contaminates different environmental matrices: air, soil, and water. Humans are continuously exposed to DEHP via dietary sources, dermal absorption, and air inhalation [1]. Consequently, DEHP has been detected in various body fluids, including urine, serum, breast milk, semen [6], and follicular fluid [7]. In the past twenty years, the European Union and the U.S. have restricted the use of several ortho-phthalates, including DEHP, in commercial products. As a result of this restriction, novel plasticizers such as di(2-ethylhexyl) adipate, 1,2-cyclohexane dicarboxylic acid diisononyl ester (DINCH), and dioctyl terephthalate (also known as di(2-ethylhexyl) terephthalate) have emerged as replacement plasticizers in PVC materials. For example, DINCH was introduced commercially in 2002 as a safer alternative to ortho-phthalate esters because of its more favorable toxicological profile compared to DEHP (https://www.efsa.europa.eu/en/efsajournal/pub/395, accessed on 12 January 2023).

The ovarian function of adult females is regulated by gonadotropins and intra-ovarian factors (e.g., steroids, growth factors, and cytokines) that regulate the follicle development and proliferation, differentiation, and steroidogenesis of granulosa cells (GC) during the process of folliculogenesis. Follicle-stimulating hormone (FSH) and luteinizing hormone (LH) are gonadotropins that are essential for proper reproduction and fertility [8]. FSH binds to the specific receptor (FSHR) located on the membrane of GC in the developing follicles and induces the proliferation and stimulation of estrogen (estrone and estradiol) production via the aromatase (CYP19A1)-mediated conversion of the androgens produced by theca cells [9]. FSH also stimulates progesterone production in GC due to the increased expression of proteins involved in cholesterol mobilization and progesterone biosynthesis, namely, steroidogenic acute regulatory protein (STAR), cholesterol side-chain cleavage enzyme (CYP11A1), and 3β-hydroxysteroid dehydrogenase (3β-HSD) [10], and it induces LH receptor formation and the LH responsiveness of GC [8]. The FSH-induced steroidogenesis is regulated by various signaling cascades, which leads to a complex pattern of gene expression in the target GC. The primary signaling cascade is the adenylyl cyclase (AC) and cyclic adenosine monophosphate/protein kinase A (cAMP/PKA) pathway, which promotes the phosphorylation of the cyclic AMP response element binding protein (CREB), a transcriptional factor that is indispensable for the expression of CYP19A1 [11]. FSH activates the phosphatidylinositol-3-kinase/AKT signaling cascade through PKA and GRB2-associated binding protein [12], which is also an obligatory pathway in the FSH-induced CYP19A1 regulation and cellular differentiation [13]. On the other hand, it has been shown that FSH can stimulate the mitogen-activated protein kinase (MAPK)/extracellular signal-regulated kinase 1/2 (ERK1/2) pathway through the activation of the epidermal growth factor (EGF) receptors (EGFR), thereby suppressing CYP19A1 expression [14] and increasing STAR, CYP11A1, and progesterone production [15].

Animal and human studies have pointed out the endocrine disrupting properties of DEHP in the female reproductive system, GC’ function, and steroidogenesis. It has been shown that the acute exposure of human GC line KGN to DEHP decreases FSH-stimulated estradiol synthesis and reduces *CYP19A1* mRNA expression [16]. DEHP decreases progesterone production [17] and increases androgen levels [7] in the primary culture of human GC. The anti-steroidogenic effect of DEHP was also supported by the results from several studies on rodent ovaries or isolated follicles, where DEHP exposure caused a decrease in serum estradiol and progesterone levels [18,19,20,21,22] or androstendione and testosterone levels [19]. It is clear that DEHP has a profound effect on steroidogenesis; however, the mechanism leading to its anti-steroidogenic effect in human GC is not well understood. One study has shown that DEHP affects estradiol production by the transactivation of the arylhydrocarbon receptor (AHR) via the peroxisome proliferator-activated receptors (PPARs) in KGN cells stimulated with FSH [16]. In rat GC, it has been shown that DEHP disrupts the basal production of estradiol and progesterone through the induction of oxidative stress [21].

In this study, we provided a mechanism by which DEHP affects steroidogenesis in FSH-stimulated human GC. Our results show that, in the primary culture of human cumulus granulosa cells (hCGC), DEHP decreases two signaling pathways activated by FSH: cAMP and ERK1/2. A decrease in the activation of these signaling cascades diminishes the expression of CYP19A1 and STAR, which, in turn, decreases the production of estradiol and progesterone in the FSH-stimulated hCGC. The concentration of 25 μM DEHP used in this study is higher than the one found in follicular fluid [7]. However, higher concentrations of DEHP in acute exposure scenarios were necessary to achieve the effect and to understand the molecular mechanism of DEHP action in human GC. For example, 50 µM DEHP was necessary to decrease the estradiol levels in the KGN cell line [16]. Moreover, 100 µM MEHP, a major DEHP metabolite, was needed to diminish the FSH-stimulated cAMP accumulation [23] and estradiol production [24] in rodent GC. On the other hand, human exposure-relevant DEHP concentrations did not change the estradiol production [7,17] and only decreased the progesterone production after 72 h [17] in human GC. We have previously shown that the repeated exposure of the human GC line HGrC1 to two environmentally relevant concentrations of DEHP (50 and 250 nM) for four weeks did not change the basal and foskolin-stimulated steroidogenesis [25]. It is possible that different GC models have different degrees of sensitivity towards DEHP, thus justifying the use of specific DEHP concentrations for the investigation of the effect and the mechanism of action of this endocrine disruptor in human GC.

## 2. Material and Methods

### 2.1. Chemicals

DEHP (analytical standard), fibronectin, bovine serum albumin fraction V, Dulbecco’s Modified Eagle’s Medium/Nutrient Mixture F-12 Ham with L-glutamine and 15 mM HEPES (DMEM/F12), penicillin, streptomycin, dimethyl sulfoxide (DMSO), TRIzol Reagent, cholera toxin, forskolin, and recombinant human EGF were obtained from Sigma-Aldrich Company (Steinheim, Germany). The purified recombinant human FSH was from Serono (Randolph, MA, USA). The STAR and phospho-ERK1/2 antibodies were from Santa Cruz Biotechnology (Dallas, TX, USA). The phospho-CREB and GAPDH antibodies were from Cell Signaling Technology (Danvers, MA, USA). The CYP19A1 antibody was from Invitrogen (Waltham, MA, USA). The horseradish peroxidase (HRP)-linked secondary anti-rabbit antibody was from Bio-Rad (Hercules, CA, USA). The Power SYBR Green PCR Master Mix was from Applied Biosystems (Applied Biosystems, Foster City, CA, USA). The cAMP, Progesterone, and Estradiol ELISA Kits were purchased from Cayman Chemicals (Ann Arbor, MI, USA).

### 2.2. Culture of Human Cumulus Granulosa Cells

The hCGC were obtained from women undergoing an *in vitro* fertilization (IVF) procedure at the Clinic for Gynecology and Obstetrics, Clinical Center of Vojvodina, Novi Sad, Serbia. For each experiment, the hCGC from 1–3 patients undergoing the IVF procedure were collected and pooled. The exclusion criteria and protocols for obtaining, isolating, and culturing hCGC have been published before [26]. The study was approved by the Ethics Committee of the Clinical Center of Vojvodina (approval number: 00-313), and signed informed consent was obtained from each participant. For all experiments, hCGC were plated in 24-well plates (0.1 × 10^6^ cells/well), except for the viability assay, where the cells were plated in 96-well plates (0.05 × 10^6^ cells/well). To analyze the effect of DEHP exposure on STAR and CYP19A1 expression and estradiol and progesterone production, the cells were either stimulated with 100 ng/mL FSH or 1μM forskolin or 10 μg/well cholera toxin alone or in a combination with 25 µM DEHP for 48 h. The same treatment plan was applied for analyzing the effect of 1 h-long DEHP exposure on cAMP levels. For analyzing the phosphorylation status of CREB and ERK1/2, the cells were either treated with 100 ng/mL FSH or 100 ng/well EGF alone or with the following combinations: 100 ng/mL FSH + 25 µM DEHP, 100 ng/mL FSH + 100 ng/well EGF, 100 ng/well EGF + 25 µM DEHP, and 100 ng/mL FSH + 100 ng/well EGF + 25 µM DEHP for 15 min.

### 2.3. Sulforhodamine B Assay

The Sulforhodamine B (SRB) assay was performed to determine whether the DEHP treatment affected the number of hCGC, as previously described [27]. Briefly, the cells were plated in a 96-well plate, treated for 48 h with DEHP, and subsequently fixed for 1 h at 4 °C by adding 50 μL of 50% (*w*/*v*) trichloroacetic acid per well. After fixation, the cells were washed five times with distilled water and stained with 50 μL of 0.4% SRB in 1% acetic acid for 30 min. After staining, the cells were washed five times with 1% acetic acid and air-dried. The stain was solubilized in 10 mM Tris (hydroxymethyl) aminomethane (pH 10.5), and the light absorption was measured using a Thermo Labsystems Multiscan EX reader set at 492 nm, with a reference wavelength at 690 nm. The samples for the analysis were run in duplicate.

### 2.4. Quantitative RT-PCR Analysis

Quantitative RT-PCR (qRT-PCR) was performed as previously described [28]. Gene-specific primer pairs for *STAR*, *CYP19A1*, and glyceraldehyde 3-phosphate dehydrogenase (*GAPDH*) were designed using the Primer Express 3.0 software (Applied Biosystems) (Table 1). Data obtained from the qRT-PCR reaction were analyzed using the comparative cycle threshold (∆∆Ct) method with an automatically adjusted fluorescence threshold (ΔRn) and then normalized to *GAPDH*. DEHP treatment had no effect on *GAPDH* expression. Samples in the qRT-PCR analysis were run in duplicate.

### 2.5. Progesterone, Estradiol, and cAMP Analysis

Progesterone and estradiol levels were estimated in the culture media using the Progesterone ELISA kit and the Estradiol ELISA kit, whereas the total cAMP levels from cells and media were extracted and measured using the cAMP ELISA kit, according to the manufacturer’s instructions. All samples in the ELSIA assays were run in duplicate.

### 2.6. Western Blot Analysis

Western blot analysis was performed as previously described [29]. The primary antibodies used were STAR (1:200), CYP19A1 (1:1000), phospho-ERK1/2 (1:3000), phospho-CREB (1:1000), and GAPDH (1:1000). After incubation with an appropriate HRP-conjugated secondary antibody (1:3000), specific immunoreactive signals were detected with the SuperSignal West Femto kit (Thermo Scientific, Waltham, MA, USA), captured digitally using the myECL imager (Thermo Scientific, Waltham, MA, USA), and quantified using the NIH ImageJ software. In each experiment, one well was used per treatment.

### 2.7. Statistical Analysis

**Figure 1:**
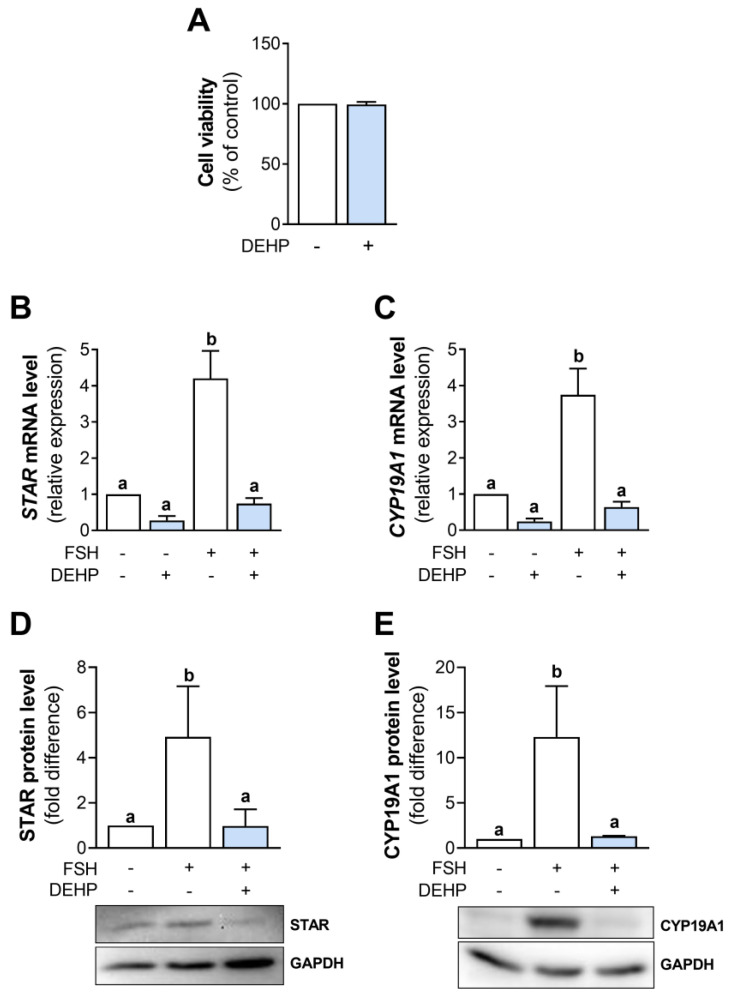
Effect of DEHP exposure on the viability of hCGC and the STAR and CYP19A1 expression in the FSH-stimulated hCGC. (**A**) Cells were treated with 25 µM DEHP for 48 h. Cell viability was assessed using the SRB assay. Results are expressed relative to the untreated control that was set as 100%. Each bar represents the mean ± SEM of three independent experiments. Cells were treated with 100 ng/mL FSH and 25 µM DEHP for 48 h, and the levels of (**B**) *STAR* and (**C**) *CYP19A1* mRNA were determined by qRT-PCR, whereas the levels of (**D**) STAR and (**E**) CYP19A1 protein were determined by Western blotting. Results are expressed relative to the untreated control that was set as 1. Representative blots are shown. Each bar represents the mean ± SEM of three to four independent experiments. Different superscript letters indicate statistically significant differences between treatment groups (*p* < 0.05).

The results are expressed as the mean ± SEM. Statistical comparisons were performed by either Student’s two-tailed t-test (Figure 1A) or one-way analysis of variance (ANOVA) and two-way ANOVA, where appropriate, followed by Tukey’s multiple comparison posthoc test (all figures starting from Figure 1B), using the GraphPad Prism 8 software package (www.graphpad.com, accessed on13 January 2023). A *p* value of <0.05 was considered significant.

## 3. Results

### 3.1. DEHP Decreases STAR and CYP19A1 mRNA and Protein Levels in FSH-Stimulated hCGC

In this study, DEHP concentration was chosen based on the previously published data indicating that 50 µM DEHP affected estradiol synthesis without changing the viability of human GC line KGN [16]. We decided to use a lower concentration of DEHP of 25 µM. The analysis shows that 25 µM DEHP did not affect the viability of hCGC after 48 h of exposure (Figure 1A).

Next, we analyzed STAR and CYP19A1 mRNA and protein levels in the FSH-stimulated hCGC following the 48h-long exposure to DEHP. The results show that DEHP did not affect the basal but decreased *STAR* (Figure 1B) and *CYP19A1* (Figure 1C) mRNA and STAR (Figure 1D) and CYP19A1 (Figure 1E) protein levels in the FSH-stimulated hCGC. DEHP also decreased the mRNA levels of *CYP11A1* and *LHCGR*, the gene encoding the LH receptor (Supplementary Figure S1).

### 3.2. DEHP Decreases Progesterone and Estradiol Levels in FSH-Stimulated hCGC

**Figure 2:**
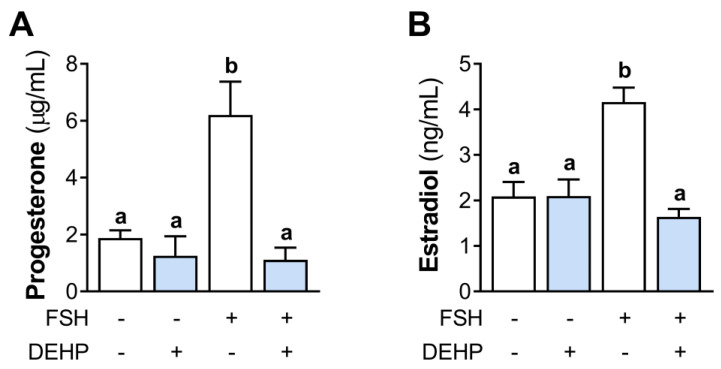
Effect of DEHP exposure on progesterone and estradiol production in the FSH-stimulated hCGC. Cells were treated with 100 ng/mL FSH and 25 µM DEHP for 48 h, and the levels of (**A**) progesterone and (**B**) estradiol in the incubation media were determined by ELISA. Each bar represents the mean ± SEM of four independent experiments. Different superscript letters indicate statistically significant differences between treatment groups (*p* < 0.05).

We next analyzed the effect of DEHP exposure on progesterone and estradiol levels in the incubation media of the FSH-stimulated hCGC. The results show that the 48h-long DEHP exposure did not affect the basal but decreased the FSH-stimulated progesterone (Figure 2A) and estradiol production (Figure 2B) in hCGC.

### 3.3. DEHP Decreases FSH- but Not Cholera Toxin- and Forskolin-Stimulated cAMP Production and CREB Phosphorylation in hCGC

**Figure 3:**
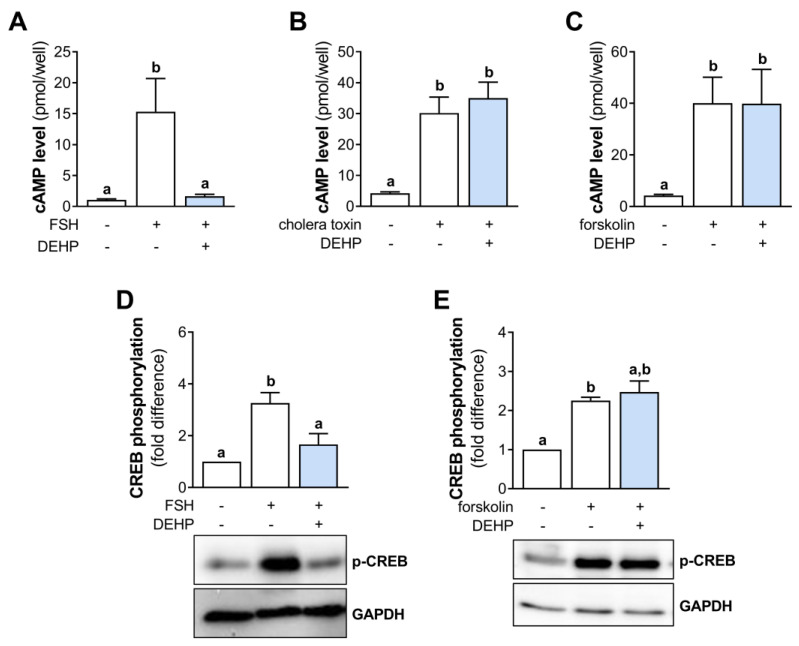
Effect of DEHP exposure on the cAMP production and CREB phosphorylation in the FSH-, cholera toxin-, and forskolin-stimulated hCGC. Cells were treated with either (**A**) 100 ng/mL FSH and 25 µM DEHP, (**B**) 10 µg/well cholera toxin and 25 µM DEHP, or (**C**) 1 μM forskolin and 25 µM DEHP for 1 h, followed by the measurement of the total cAMP levels using ELISA. For Western blot analysis, hCGC were treated with either (**D**) 100 ng/mL FSH and 25 µM DEHP or (**E**) 1 μM forskolin and 25 µM DEHP for 15 min. Results are expressed relative to the untreated control that was set as 1. Representative blots are shown. Different superscript letters indicate statistically significant differences between treatment groups (*p* < 0.05).

In an attempt to clarify the mechanism of DEHP action in hCGC, we investigated the cAMP signaling. First, we measured the cAMP levels in the FSH-stimulated hCGC following the 1 h-long DEHP exposure. The results indicate that FSH increases the cAMP production in hCGC, which was prevented by the addition of DEHP (Figure 3A). To avoid the receptor-mediated cAMP accumulation, we next stimulated hCGC with the cholera toxin (which binds and activates the Gsα, thereby leading to a continuous stimulation of AC and the production of cAMP) and forskolin (which activates AC and increases the cAMP production). In these experiments, DEHP exposure did not affect the cholera toxin- (Figure 3B) and forskolin-induced (Figure 3C) cAMP production in hCGC. Furthermore, we examined the effect of DEHP exposure on the phosphorylation of CREB in the FSH- or forskolin-stimulated hCGC. The results show that DEHP decreased FSH-induced CREB phosphorylation (Figure 3D) but had no effect on CREB phosphorylation when hCGC were stimulated with forskolin (Figure 3E).

### 3.4. DEHP Does Not Affect Cholera Toxin- and Forskolin-Stimulated STAR and CYP19A1 mRNA Levels in hCGC

**Figure 4:**
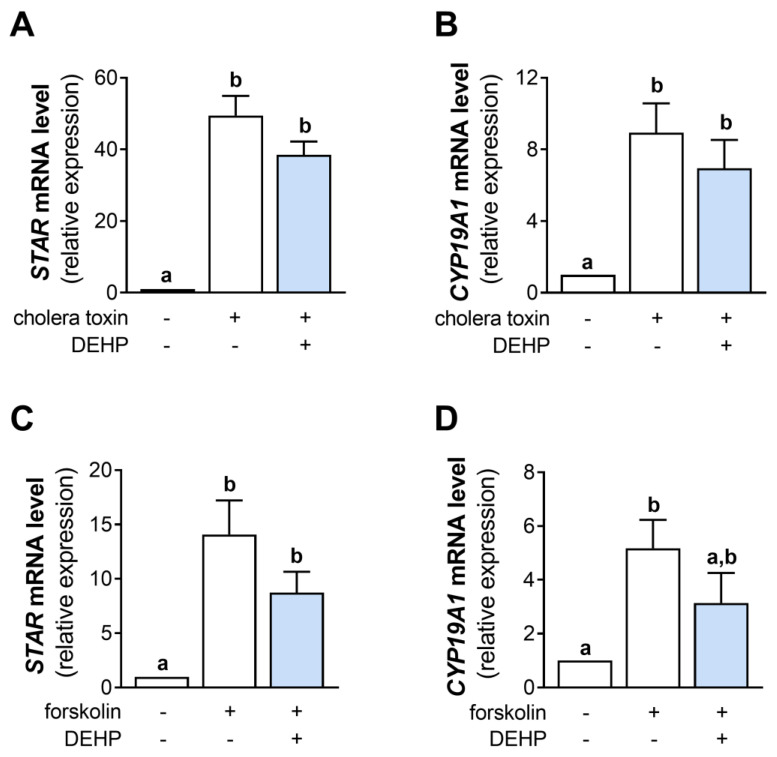
Effect of DEHP exposure on the cholera toxin- and forskolin-stimulated *STAR* and *CYP19A1* mRNA expression in hCGC. Cells were treated either with 10 µg/well cholera toxin and 25 µM DEHP for 48 h, and the levels of (**A**) *STAR* and (**B**) *CYP19A1* mRNA were determined by qRT-PCR, or with 1 μM forskolin and 25 µM DEHP for 48 h, and the levels of (**C**) *STAR* and (**D**) *CYP19A1* mRNA were determined by qRT-PCR. Results are expressed relative to the untreated control that was set as 1. Each bar represents the mean ± SEM of four independent experiments. Different superscript letters indicate statistically significant differences between treatment groups (*p* < 0.05).

Since DEHP did not change the cAMP production in the cholera toxin- and forskolin-stimulated hCGC, we next analyzed the mRNA levels of *STAR* and *CYP19A1*. The results show that the 48 h-long DEHP exposure did not affect the cholera toxin-stimulated *STAR* (Figure 4A) and *CYP19A1* (Figure 4B) mRNA levels and the forskolin-stimulated *STAR* (Figure 4C) and *CYP19A1* mRNA levels (Figure 4D) in hCGC.

### 3.5. DEHP Does Not Affect Cholera Toxin- and Forskolin-Stimulated Hormone Production in hCGC

**Figure 5:**
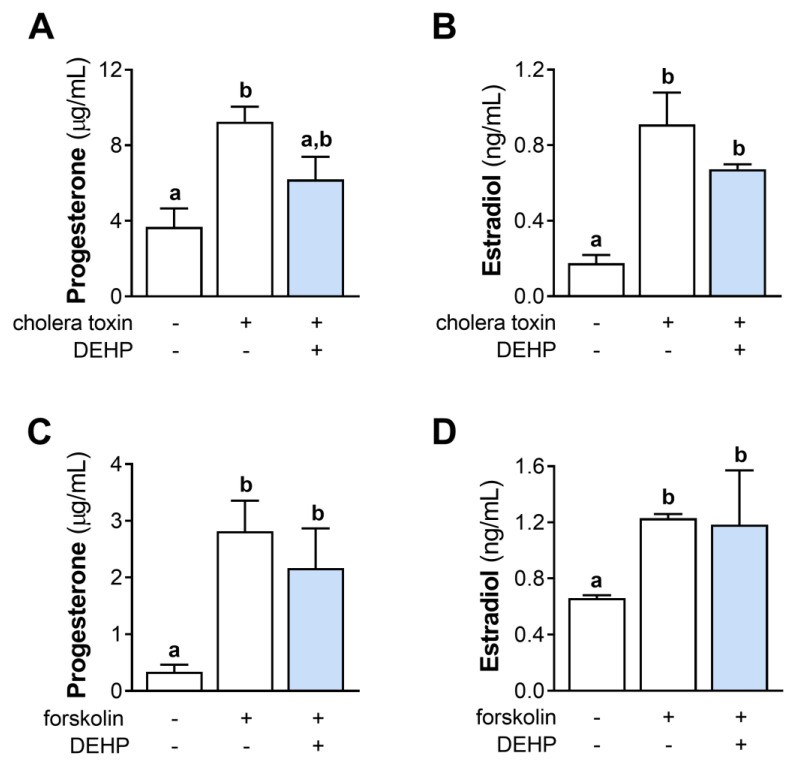
Effect of DEHP exposure on the cholera toxin- and forskolin-stimulated progesterone and estradiol production in hCGC. Cells were treated either with 10 µg/well cholera toxin and 25 µM DEHP for 48 h, and the levels of (**A**) progesterone and (**B**) estradiol in the incubation media were determined by ELISA, or with 1 μM forskolin and 25 µM DEHP for 48 h, and the levels of (**C**) progesterone and (**D**) estradiol in the incubation media were determined by ELISA. Each bar represents the mean ± SEM of four independent experiments. Different superscript letters indicate statistically significant differences between treatment groups (*p* < 0.05).

We further evaluated the effect of the 48 h-long DEHP exposure on the cholera toxin- and forskolin-stimulated steroid hormones’ production in hCGC. The results show that DEHP exposure did not affect the cholera toxin-stimulated progesterone (Figure 5A) and estradiol (Figure 5B) production, as well as the forskolin-stimulated progesterone (Figure 5C) and estradiol (Figure 5D) production in hCGC.

### 3.6. EGF Prevents the DEHP-Mediated Inhibition of ERK1/2 Phosphorylation in FSH-Stimulated hCGC

**Figure 6:**
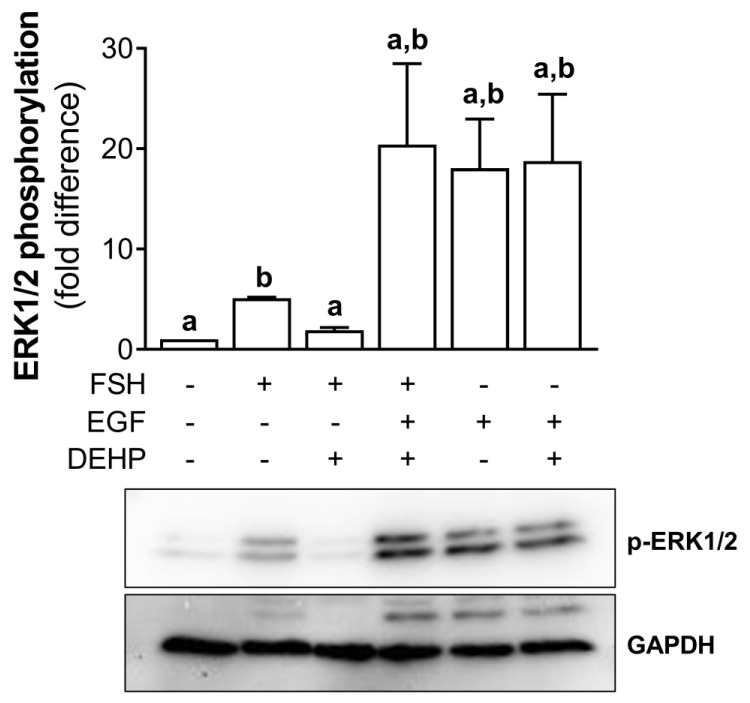
Effect of DEHP exposure on ERK1/2 phosphorylation in the FSH-, FSH+EGF-, and EGFstimulated hCGC. Cells were treated with either 100 ng/mL FSH and 25 µM DEHP, 100 ng/mL FSH + 100 ng/well EGF and 25 µM DEHP, or 100 ng/well EGF and 25 µM DEHP for 15 min, and the level of ERK1/2 phosphorylation was analyzed by Western blotting. Results are expressed relative to the untreated control that was set as 1. Representative blots are shown. Each bar represents the mean ± SEM of three independent experiments. Different superscript letters indicate statistically significant differences between treatment groups (*p* < 0.05).

In addition, we analyzed the involvement of ERK1/2 in DEHP-mediated anti-steroidogenic action in the FSH-stimulated hCGC. First, we showed that DEHP decreases the FSH-stimulated ERK1/2 phosphorylation in hCGC. Then, we treated the cells with EGF to override the DEHP-mediated inhibition of ERK1/2 observed in the FSH-stimulated hCGC. The addition of EGF to the FSH-stimulated and DEHP-exposed hCGC prevented the inhibition of ERK1/2, thereby showing that the ERK1/2 signaling was restored. EGF alone provokes a strong stimulation of ERK1/2, which was not affected by the addition of DEHP (Figure 6).

### 3.7. EGF Prevents the DEHP-Mediated Inhibition of STAR and CYP19A1 mRNA Expression in FSH-Stimulated hCGC

**Figure 7:**
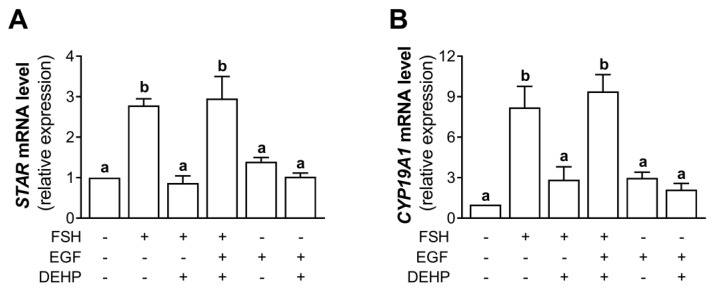
Effect of DEHP exposure on *STAR* and *CYP19A1* mRNA expression in the FSH-, FSH+EGF-, and EGF-stimulated hCGC. Cells were treated with either 100 ng/mL FSH and 25 µM DEHP, 100 ng/mL FSH + 100 ng/well EGF and 25 µM DEHP, or 100 ng/well EGF and 25 µM DEHP for 48 h, and the levels of (**A**) *STAR* and (**B**) *CYP19A1* mRNA were determined by qRT-PCR. Results are expressed relative to the untreated control that was set as 1. Each bar represents the mean ± SEM of three independent experiments. Different superscript letters indicate statistically significant differences between treatment groups (*p* < 0.05).

Using the experimental approach described in Section 3.7, we analyzed the mRNA levels of *STAR* and *CYP19A1*. The results indicate that the addition of EGF prevented the DEHP-mediated inhibition of *STAR* (Figure 7A) and *CYP19A1* (Figure 7B) mRNA levels in the FSH-stimulated hCGC.

### 3.8. EGF Prevents the DEHP-Mediated Inhibition of Progesterone and Estradiol Production in FSH-Stimulated hCGC

**Figure 8:**
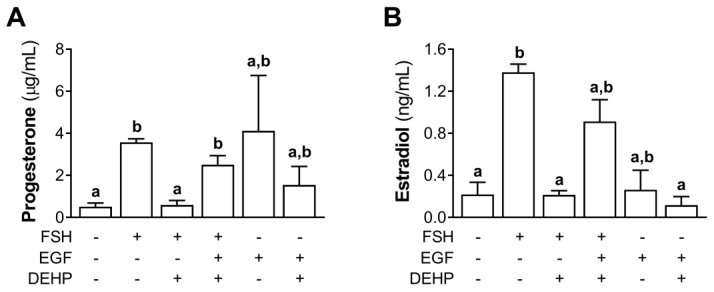
Effect of DEHP exposure on progesterone and estradiol production in the FSH-, FSH+EGF-, and EGF-stimulated hCGC. Cells were treated with either 100 ng/mL FSH and 25 µM DEHP, 100 ng/mL FSH + 100 ng/well EGF and 25 µM DEHP, or 100 ng/well EGF and 25 µM DEHP for 48 h, and the levels of (**A**) progesterone and (**B**) estradiol were determined by ELISA. Each bar represents the mean ± SEM of three independent experiments. Different superscript letters indicate statistically significant differences between treatment groups (*p* < 0.05).

We also measured the progesterone and estradiol levels in the EGF- and FSH-stimulated hCGC exposed to DEHP for 48 h. The results show that EGF prevented the DEHP-mediated inhibition of progesterone (Figure 8A) and estradiol (Figure 8B) production in the FSH-stimulated hCGC.

## 4. Discussion

In this study, we investigated the effect and the mechanism of DEHP action in the FSH-stimulated hCGC. We have shown that DEHP decreases the FSH-stimulated expression of CYP19A1 and STAR and lowers the production of estradiol and progesterone. The mechanistic experiments revealed that the cAMP and ERK1/2 signaling pathways mediate the anti-steroidogenic action of DEHP in the FSH-stimulated hCGC.

The effect of DEHP on basal steroidogenesis in human GC was investigated in several studies. It has been shown that DEHP can decrease the basal production of androgens and the expression of CYP19A1 and CYP17A1 in the primary culture of human GC and human GC line KGN [7] and the progesterone production in the primary culture of human GC [17]. To date, only one study has shown that DEHP can affect hormone-stimulated steroidogenesis by decreasing the FSH-induced estradiol production in KGN cells without affecting the basal production of this steroid [16]. Here, we have also shown that DEHP did not change the basal production of estradiol and progesterone but decreased the production of these steroids after the stimulation of hCGC with FSH. The reduction in the levels of both steroid hormones was accompanied by a decrease in the expression of the key enzymes involved in steroid production, namely, *CYP19A1* and *STAR*. These results provide an insight into the mechanism by which DEHP reduces the production of steroid hormones in the FSH-stimulated hCGC. It appears that DEHP affects the expression of two key steroidogenic enzymes to lower the production of estradiol and progesterone. The mechanism of the DEHP-mediated decrease in steroid hormones’ production in the FSH-stimulated hCGC described in this study is somewhat different from the one suggested by Ernst and co-workers. In their study, the authors did not observe changes in *CYP19A1* mRNA expression in DEHP- and FSH-treated KGN cells; however, DEHP treatment caused an increase in the expression of *AHR* and *PPARs*. The authors suggested that the increase in the metabolism of estradiol and the disruption of the PPAR signaling could be the potential mechanism of the DEHP-induced decrease in estradiol levels [16].

The inhibitory effect of DEHP on *CYP19A1* and *STAR* mRNA levels prompts further analysis of the signaling pathways involved in this DEHP-mediated process. In our study, DEHP diminished the FSH-stimulated cAMP production and CREB phosphorylation. Since the cAMP signaling pathway plays an important role in the expression of CYP19A1 and STAR in GC stimulated with FSH [15], lower cAMP production in DEHP-exposed human GC could explain the observed decrease in the expression of *CYP19A1* and *STAR*. Further confirmation that cAMP could be the potential mechanism for the DEHP-mediated decrease in *CYP19A1* and *STAR* mRNA levels was obtained in experiments with cholera toxin and forskolin. In the cholera toxin- and forskolin-stimulated hCGC, DEHP was not able to diminish the cAMP levels, indicating that the cholera toxin and forskolin treatment affected the ability of this endocrine disruptor to prevent the expression of *CYP19A1* and *STAR*. Moreover, DEHP was not able to reduce the synthesis of estradiol and progesterone in both cholera toxin- and forskolin-stimulated hCGC. By overriding the effect of DEHP on cAMP production, we were able to rescue the steroidogenesis, thus highlighting that cAMP could be one of the DEHP targets in hCGC stimulated with FSH. The experiments with forskolin and cholera toxin also suggest that DEHP does not interfere with the activation of AC or the Galpha subunit to decrease the cAMP level in hCGC stimulated with FSH. Moreover, it appears that DEHP does not affect any of the cAMP downstream effectors, since the activation of the cAMP pathway by forskolin and cholera toxin stimulates steroidogenesis in DEHP-exposed hCGC. These data suggest that DEHP possibly interferes with the activity of the FSH receptor on the cell membrane; however, this assumption should be confirmed by further experimental studies.

The potential role of the cAMP pathway in the anti-steroidogenic action of DEHP in human GC has not been shown before. Limited information regarding the role of the cAMP pathway in the anti-steroidogenic action of DEHP was obtained using mono-2-ethylhexyl phthalate (MEHP), a major DEHP metabolite, and rodent GC; however, the results were somewhat inconsistent. While some authors have shown that cAMP could be the target of DEHP action, leading to lower progesterone levels in the FSH-stimulated rat GC exposed to MEHP [18,23], others have shown that MEHP could decrease the estradiol production [24] or CYP19A1 expression [30] independently of the FSH-cAMP stimulation. Moreover, it has been shown that MEHP can stimulate basal steroidogenesis through a cAMP- and STAR-independent mechanism [31]. In addition, it was proposed that MEHP can activate PPARs to decrease the CYP19A1 expression in rat GC [18]. The involvement of PPARs in the anti-estrogenic action of DEHP could also be a possible mechanism of DEHP action in human GC for the following reasons: (i) DEHP metabolism to MEHP has been shown in human GC, and (ii) DEHP affects the expression of PPARα and PPARβ in human GC [16]. Therefore, the proposed target of DEHP anti-steroidogenic action in human GC can include two points in the steroid biosynthetic pathway, cAMP and PPARs, as suggested elsewhere [18].

Besides changes in the FSH-stimulated cAMP pathway in DEHP-exposed human GC, we also noticed that DEHP decreases the FSH-induced ERK1/2 phosphorylation. Since ERK1/2 plays a critical role in the regulation of CYP19A1 [14] and STAR [15] in the FSH-stimulated GC, a DEHP-mediated decrease in ERK1/2 activity could be responsible for the observed decrease in the expression of *CYP19A1* and *STAR* in hCGC stimulated with FSH. Further reinforcement for the role of ERK1/2 in the anti-steroidogenic action of DEHP was obtained in the experiments with EGF. The stimulation of human GC with EGF restored the ERK1/2 activity and prevented the observed decline in the expression of *CYP19A1* and *STAR* and the production of steroid hormones in the DEHP-exposed and FSH-stimulated human GC. These data clearly show that ERK1/2 could also be the target of DEHP action in human GC. This represents a novel finding, as the connection between DEHP and ERK1/2 in human GC has not been reported before. It is known that DEHP can change the ERK1/2 activity in hepatocytes [32], rat testes [33], and endometrial stroma cells [34].

The remaining question is whether DEHP activates cAMP and ERK1/2 in a linear pathway or whether these two signaling cascades are affected independently in the DEHP-exposed and FSH-stimulated human GC. In the FSH-stimulated GC, ERK1/2 can be activated via the PKA-dependent inactivation of MAPK phosphatase 3 (MKP3) [35] or through the transactivation of the EGFR [14,36]. The EGFR transactivation pathway is supported by the data indicating that FSH can stimulate amphiregulin (AREG) expression [15,37] and its release [38] and that the cAMP signaling pathway is involved in the expression of AREG in FSH-stimulated GC [15,37]. Since DEHP decreases cAMP production in the FSH-stimulated hCGC, we can assume that the cAMP–EGFR signaling pathway could be a possible mediator of DEHP action in the FSH-stimulated hCGC. By decreasing the cAMP levels in FSH-stimulated hCGC, DEHP diminishes the expression or the release of the EGFR growth factors, which affects the activation of EGFR, thus lowering ERK1/2 phosphorylation. This notion not only indicates that a linear cAMP–ERK1/2 signaling pathway could be the mediator of DEHP anti-steroidogenic action in the FSH-stimulated human GC but also provides the possible mechanism by which DEHP decreases the ERK1/2 activity in hCGC stimulated with FSH. However, we are not sure which of the cAMP-dependent pathways of ERK1/2 activation is affected by DEHP exposure (the PKA-dependent inactivation of MKP3 or the transactivation of the EGFR). We analyzed the expression of *AREG* and noticed that DEHP did not affect the mRNA level of this EGFR ligand in the FSH-stimulated hCGC (data not shown); however, more experiments are needed to shed more light on the mechanism by which DEHP affects the cAMP and ERK1/2 pathway in the FSH-stimulated hCGC.

## 5. Conclusions

In this study, we have shown novel findings indicating that DEHP can decrease the activity of the cAMP and ERK1/2 signaling pathway, leading to diminished estradiol and progesterone biosynthesis in the FSH-stimulated hCGC. This knowledge could have a significant impact on our understanding of how DEHP affects human female reproductive health.

## Figures and Tables

**Table 1:** Primer sequences used for the qRT-PCR analysis.

Forward and Reverse Primer	mRNA (Human)
5’-CGAAGAACCACCCTTGAGAGAA-3’ 5’-AGCATTGTTTCCTGGCAAATG-3’	*STAR*
5’-CGTGGCTGTGCAGGAAAGT-3’ 5’-CAACACACTGTCCTTGCAATGTC-3’	*CYP19A1*
5’-CAAGGCTGTGGGCAAGGT-3’ 5’-GGAAGGCCATGCCAGTGA-3’	*GAPDH*

## Data Availability

The data that support this study will be shared upon reasonable request to the corresponding author.

## Supplementary Materials

The following supporting information can be downloaded at: https://www.mdpi.com/article/10.3390/cells12030398/s1, Figure S1: Effect of DEHP exposure on *CYP11A1* and *LHCGR* mRNA expression in the FSH-stimulated hCGC; Figure S2: Standard curves obtained from the ELISA for (A) cAMP, (B) progesterone, and (C) estradiol; Table S1. Primer sequences used for the qRT-PCR analysis.
