# Supplementary material for: DEHP Decreases Steroidogenesis through the cAMP and ERK1/2 Signaling Pathways in FSH-Stimulated Human Granulosa Cells
Source: Cells. 2023 Jan 22;12(3):398. doi: 10.3390/cells12030398 (PMC9913623; doi:10.3390/cells12030398)
Supplement: Supplementary file 1 [file cells-12-00398-s001.zip › cells-2149463-supplementary.pdf]

DEHP Decreases Steroidogenesis through the cAMP and ERK1/2 Signaling Pathways in FSH-Stimulated Human Granulosa Cells

Biljana Tesic[1], Dragana Samardzija Nenadov[1], Tamara Tomanic[1], Svetlana Fa Nedeljkovic[1], Stevan Milatovic[2], Bojana Stanic[1], Kristina Pogrmic-Majkic[1]*, Nebojsa Andric[1]

[1] Department of Biology and Ecology, Faculty of Sciences, University of Novi Sad, 21000 Novi Sad, Serbia

[2] Clinical Center of Vojvodina, Clinic for Gynecology and Obstetrics, Faculty of Medicine, University of Novi Sad, 21000 Novi Sad, Serbia

*Corresponding author: Kristina Pogrmic-Majkic, Ph.D.

Department of Biology and Ecology, Faculty of Sciences, University of Novi Sad

Trg Dositeja Obradovica 2, 21000 Novi Sad, Serbia

phone: +381 21 485 2675, email: kristina.pogrmic@dbe.uns.ac.rs

ORCID: 0000-0001-9274-7723

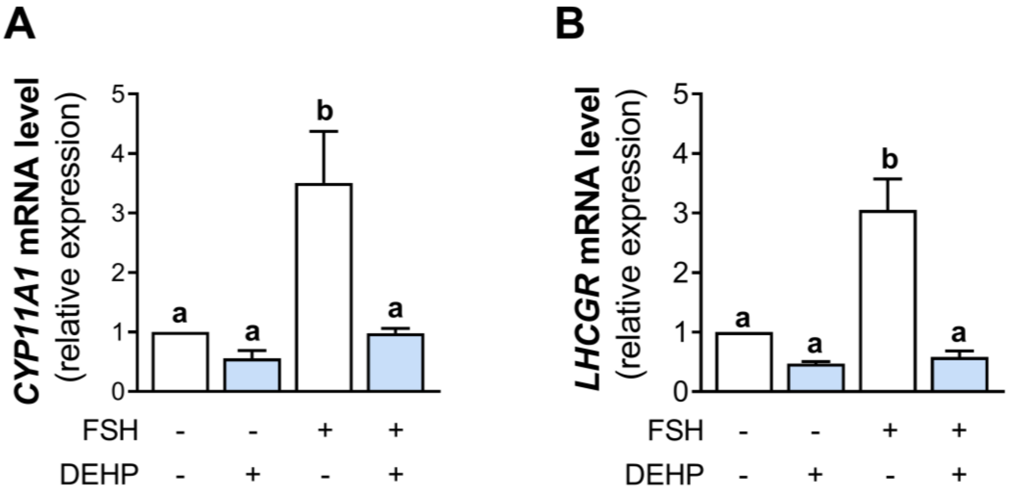

Figure S1. Effect of DEHP exposure on *CYP11A1* and *LHCGR* mRNA expression in the FSH-stimulated hCGC. Cells were treated with 100 ng/mL FSH and 25 µM DEHP for 48 h and the levels of **(A)** *CYP11A1* and **(B)** *LHCGR* mRNA were determined by qRT-PCR. Results were expressed relative to the untreated control that was set as 1. Each bar represents the mean ± SEM of 3 independent experiments. Different superscript letters indicate statistically significant differences between treatment groups (p <0.05).

Table S1. Primer sequences used for the qRT-PCR analysis.

**Forward and reverse primer**	**mRNA (human)**
5'-GGAGACGGGCACACACAAA-3'<br>5'-CCCTGTAAATCGGGCCATACT-3'	*CYP11A1*
5'-GGGCCGAAAACCTTGGAT-3'<br>5'-TGAATGGACTCTAGGCCATAGCT-3'	*LHCGR*
5'-CAAGGCTGTGGGCAAGGT-3'<br>5'-GGAAGGCCATGCCAGTGA-3'	*GAPDH*

Gene-specific primer pairs for *CYP11A1*, *LHCGR*, and *GAPDH* were designed using the Primer Express 3.0 software (Applied Biosystems).

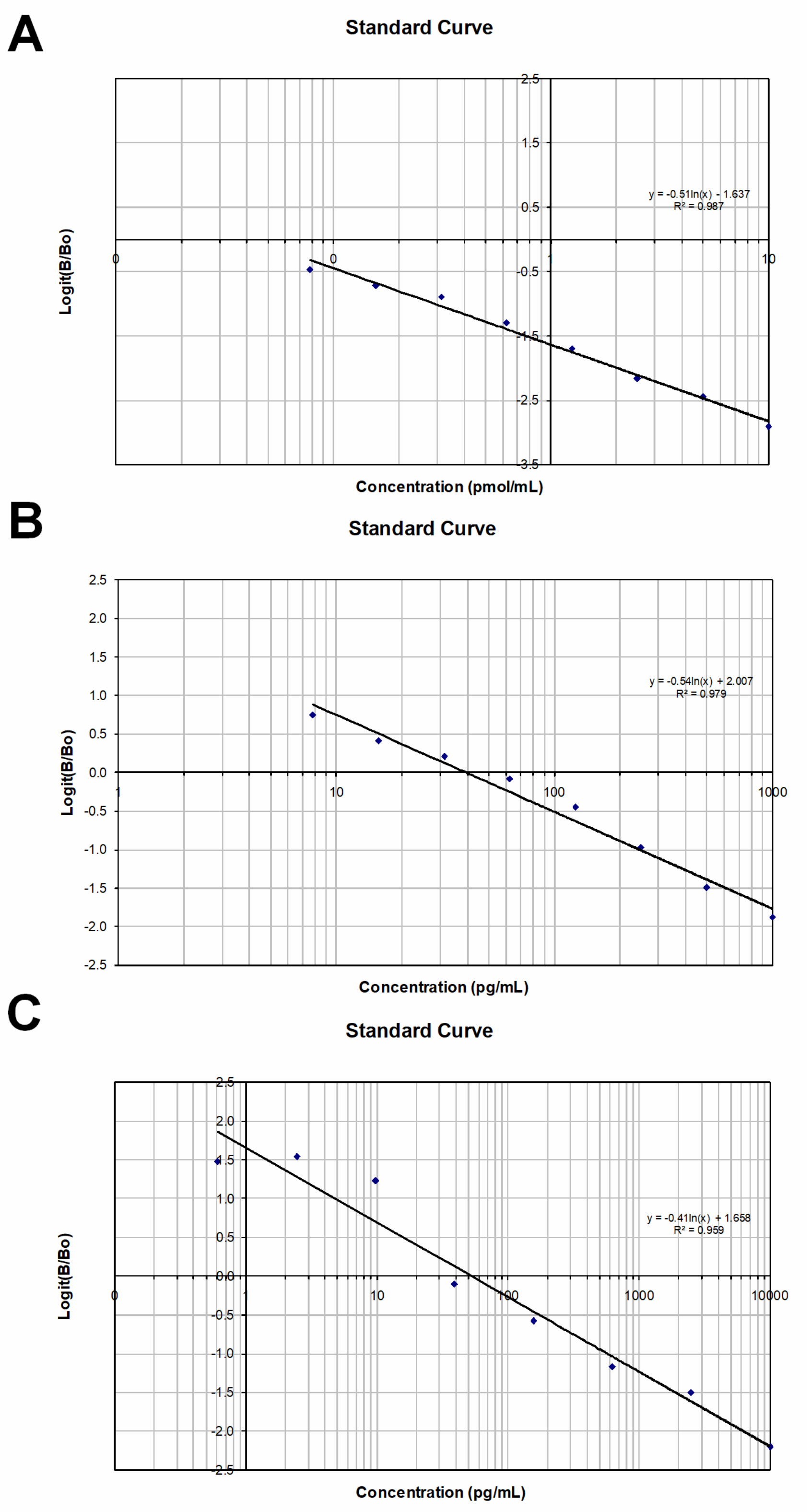

Figure S2. Standard curves obtained from the ELISA for **(A)** cAMP, **(B)** progesterone, and **(C)** estradiol.
